# Protocol for the preparation, execution, and automated analysis via IntelliR of IntelliCage-based mouse experiments

**DOI:** 10.1016/j.xpro.2025.104246

**Published:** 2025-12-03

**Authors:** Martin Hindermann, Vinicius Daguano Gastaldi, Justus Wilke, Anja Ronnenberg, Sahab Arinrad, Sabine Kraus, Hannelore Ehrenreich

**Affiliations:** 1 Clinical Neuroscience, Max Planck Institute for Multidisciplinary Sciences, City Campus, 37075 Göttingen, Germany; 2 University of Göttingen, 37073 Göttingen, Germany; 3 Experimental Medicine, Department of Psychiatry and Psychotherapy, Central Institute of Mental Health, Medical Faculty Mannheim, Heidelberg University, J 5, 68159 Mannheim, Germany

**Keywords:** Neuroscience, cognitive Neuroscience, behavior

## Abstract

The rapidly evolving field of rodent behavior research led to the development of the IntelliCage, enabling observer-independent experiments with mice. Here, we present a detailed protocol for IntelliCage-based automated cognitive profiling of mice that navigates users through all experimental steps. In addition to instructions on how to prepare, execute, and analyze the experiment, we provide empirically informed recommendations and notes that may help new users plan robust and reproducible IntelliCage-based experiments.

For complete details on the use and execution of this protocol, please refer to Gastaldi et al.^1^

## Highlights

• Instructions for mouse preparations for IntelliCage-based experiments
• Procedures for preparation of experimental design and IntelliCage
• Guidance on executing and controlling IntelliCage-based experiments
• Steps for the analysis of IntelliCage data by the automated pipeline IntelliR

## Abstract (editor highlights)

Publisher’s note: Undertaking any experimental protocol requires adherence to local institutional guidelines for laboratory safety and ethics.

## Graphical abstract

**Figure:**
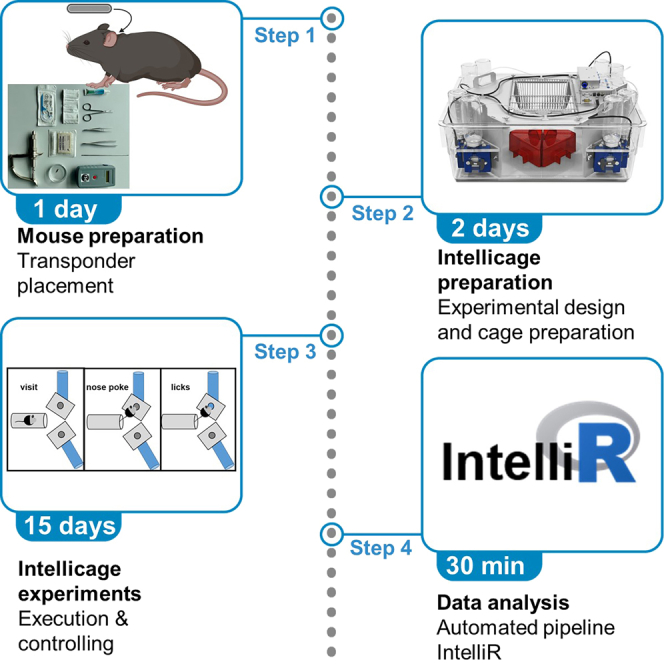

## Before you begin

The increasing demand on behavioral research on mice in combination with challenges of established behavior tests in regard to excessive handling, social isolation as well as testing the animals during their resting phase, led to the development of the IntelliCage (TSE Systems, Berlin, Germany),^2^ a well-established system, also previously utilized in our group.^2^^,^^3^^,^^4^ Fitting into a standard Makrolon type IV rodent cage (20.5 × 55 × 38.5 cm), the system allows housing of up to 16 mice, enabling behavioral testing within a social and therefore stress-reduced context. Regarding the spatial requirements per mouse, please check your local regulatories. The system consists of four computer-controlled operant training chambers, one in each corner of the cage. Mice are able to enter each operant chamber through a tube which is equipped with a radio-frequency identification (RFID) antenna, individually identifying the visiting animal by the afore implanted transponder. Within each chamber, there are two operant conditioning walls, each equipped with sensor beams close to the surface, enabling the detection of a visiting mouse’s nosepoke. In the center of each operant conditioning wall is a computer-controlled door, giving access to the tip of a water bottle behind it. Furthermore, each bottle is equipped with a “lickometer”, measuring the individual number of licks. Additionally, there are three light emitting diodes (LEDs) above each door which can be individually controlled, allowing computer-controlled display of light in three different intensities and four colors (red, blue, green, yellow). Each operant conditioning chamber also contains an outlet to deliver air-puffs as aversive stimuli, which can be delivered at up to 2 bar, depending on your valves settings.^5^ The three basic parameters on mouse behavior provided by the IntelliCage system are visits (RFID & presence in tube), nosepokes (sensor beams, operant wall), and licks (liquid consumption, tip of bottles). Furthermore, the system provides continuously collected data on environmental factors, such as ambient temperature and light intensity.

### Innovation

Standardizing behavioral testing in freely moving mice remains a critical challenge despite the reproducibility gains achieved with the IntelliCage system. The large number of studies using this platform has led to substantial variability in data analysis approaches.^2^^,^^3^ To harmonize experimental design and analysis, we introduce a detailed protocol that combines IntelliCage task implementation with IntelliR, an automated, open-source analysis pipeline. This protocol enables the assessment of spatial learning and memory, episodic-like memory, and working memory, including their respective reversal phases to evaluate cognitive flexibility in mice, by employing the provided designer files for these tasks. Moreover, we describe how to execute IntelliR, a free and open-source automated pipeline to analyze the behavior of up to four different groups, allocate task-specific errors including statistics, and subsequently calculate the Cognition Index, allowing performance comparison across varying tasks.^1^ Notably, besides enabling the automated pipeline analysis of spatial learning and memory, episodic-like memory, and working memory in mice being tested in the IntelliCage, this protocol can be adapted to a variety of mouse models, different tasks according to the individual research question, as well as adjusted analysis through IntelliR.

### Protocol overview

**Figure 1:**
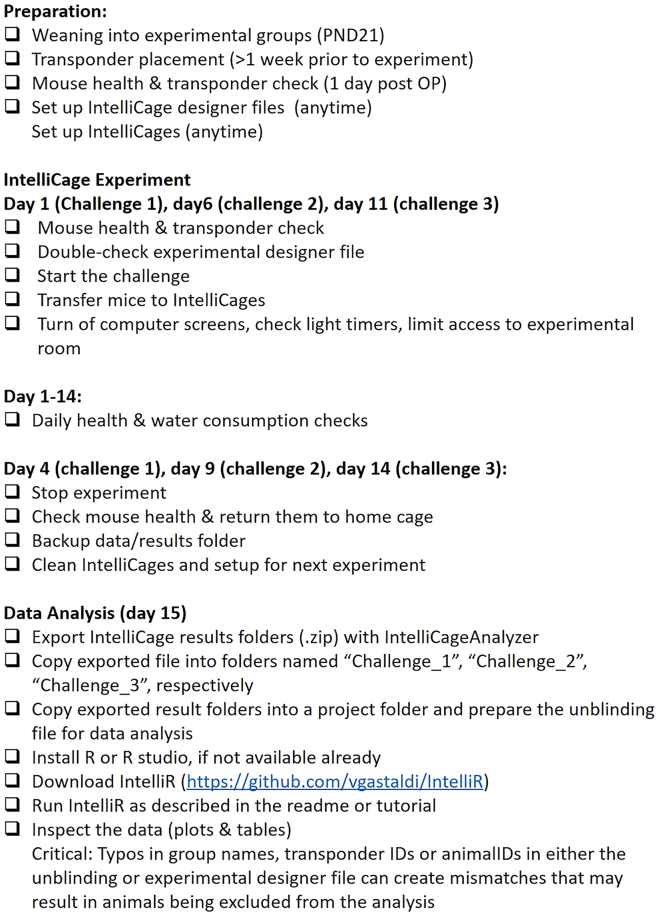
IntelliR checklist

This protocol provides a detailed instruction on how to prepare mice and the IntelliCage for correspondent experiments. Furthermore, we demonstrate how to execute and control the running trials, as well as analyzing the acquired raw data via IntelliR. For a detailed description and validation on the previously designed experiments, please see Gastaldi, Hindermann & Wilke et al. 2025. A comprehensive overview of the workflow is provided in the IntelliR checklist (Figure 1).

### Institutional permissions

Using this protocol for automated cognitive profiling of mice requires prior approval from relevant institutional and/or national animal regulatory authorities. Biometric and experimental planning should be performed in accordance with the ARRIVE guidelines. In our experience, the expected experimental burden of the provided protocol is minimal in wildtype mice, if the provided termination criteria are followed.

All animal procedures described in this protocol were approved by the local animal care and use committee (LAVES, Niedersächsisches Landesamt für Verbraucherschutz und Lebensmittelsicherheit, Oldenburg, Germany, license number: 33.19-42502-04-18/2803).

### Mouse preparation

**Timing: 1 day**

This section will prepare mice for the IntelliCages and can be performed way in advance of the behavioral experiments.

1. Upon weaning, all mice are housed in groups already planned for IntelliCage experiments.

***Note:*** If unavoidable, females can be grouped together at a later time. Environmental housing conditions should be a consistent humidity (∼50%) and temperature (∼22°C), as well as a 12 h light-dark cycle with water and food *ad libitum*. We recommend the standard housing for IntelliCages and wood chipped bedding (refer to local regulatories), which should be enough to cover the cage’s floor (approx. 3L). Avoid bedding in conditioning corners, as it can block doors.

**CRITICAL:** Housing in stable groups from weaning onwards will reduce the chances of infighting, particularly in male mice. In our experience, mice are best weaned at PND21 and housed in groups of up to 16 mice in type IV 55 × 38.5 × 20.5 cm (rat) cages. Published studies reported on using both sexes in the Intellicage,^4^^,^^6^ different mouse strains and mutants^4^^,^^7^ as well as wild-caught mice of both sexes.^8^

**Figure 2:**
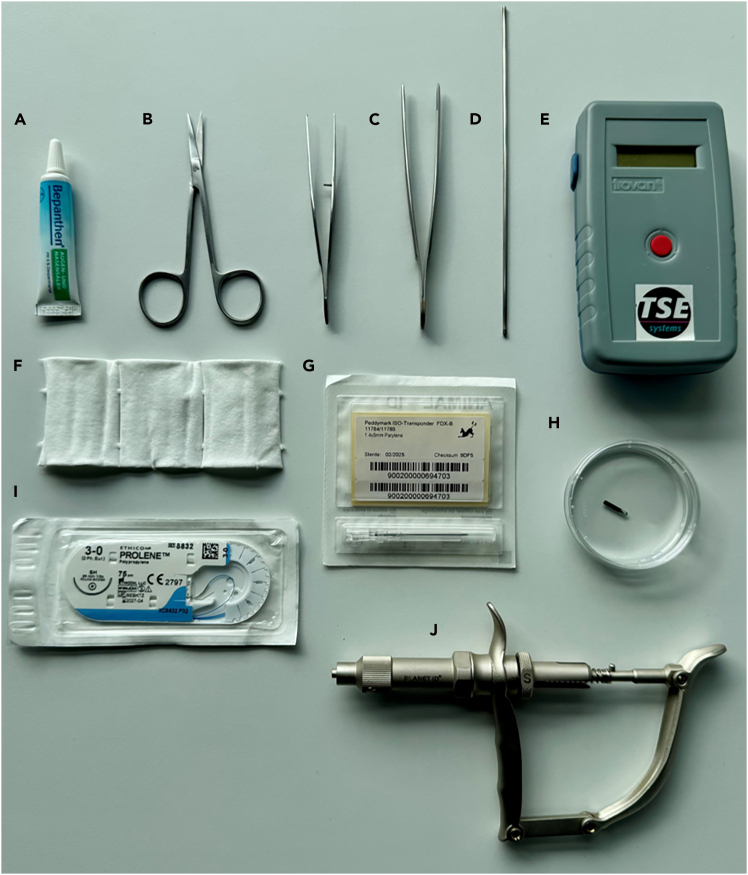
Tools and equipment for mouse preparation (A) Eye protection salve, (B) Scissors, (C) Forceps, (D) Bulb-headed probe, (E) Transponder scanner by TSE, (F) Cotton pads, (G) Transponder originally packed, (H) Re-usable transponder in ringer solution, (I) Suture material polypropylene 3-0, (J) Injector with needle.

2. Transponder placement.
  a. Via disposable injector with needle for first-time usage of transponders.
    i. Prepare working area for intervention (anesthesia, ethanol, cotton pad, forceps, injector with needle, transponder, transponder scanner, eye protection salve, heating pad, Figure 2).
    ii. Prepare new home cage and place it on a heating pad (∼37°C) to avoid hypothermia after intervention.
    iii. Note transponder ID from package together with mouse ID and load transponder into injector.
    iv. Anesthetize mouse and position it onto its belly. If injection anesthesia, i.e., Avertin or Ketamine, is used, apply protection salve onto eyes.
    v. Disinfect skin at the neck with ethanol-soaked cotton pad.
    vi. Lift up skin of the neck with forceps and use injector for subcutaneous transponder placement.
    vii. Scan freshly placed transponder and compare ID with notes.
    viii. End anesthesia and gently place mouse into its new home cage.
  b. Via incision and suture for re-usage of transponders.
    i. Prepare working area for intervention (anesthesia, ethanol, cotton pad, forceps, scissors, bulb-headed probe, suture material (polypropylene 3-0), transponder, transponder scanner, eye protection salve, heating pad, Figure 2).
    ii. Prepare new home cage and place it on a heating pad (∼37°C) to avoid hypothermia after intervention.
    iii. Put clean transponder in small dish with ringer solution or buffer.
    iv. Scan transponder ID and note together with mouse ID.
    v. Anesthetize mouse and position it onto its belly. If injection anesthesia, i.e., Avertin or Ketamine, is used, apply protection salve onto eyes.
    vi. Disinfect skin at the neck with ethanol-soaked cotton pad.
    vii. Lift up skin of the neck with forceps and do a small incision (∼3 mm) with scissors.
    viii. Use bulb-headed probe to create a small pocket between skin and muscle and place transponder within.
    ix. Scan freshly placed transponder and compare ID with notes.
    x. Close incision with suture material.
    xi. End anesthesia and gently place mouse into its new home cage.

***Note:*** The duration of the actual transponder placement per mouse may vary depending on practical experience and chosen approach (2a or 2b). In our experience, it can be done within ∼30 s (method 2a) up to 5 min (method 2b). The full duration of anesthesia depends on the chosen method, which has to be in accordance with local regulatories.

**CRITICAL:** Be aware that the mice might try to manipulate each other’s injection sites, which might cause the loss of a placed transponder. We recommend to check for the transponder whenever possible before the start of the experiment (e.g. handling).

### IntelliCage preparation

**Timing: 2 days**

This section will ensure proper IntelliCage functioning prior to the behavioral experiments.

**Figure 3:**
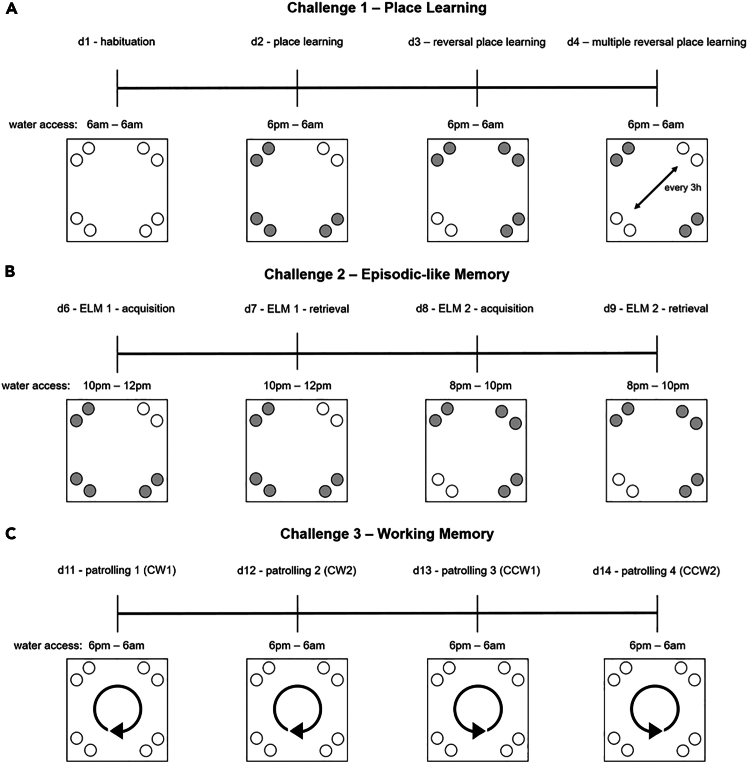
Descriptive overview of the programmed challenges The three provided challenges include (A) habituation and place learning with (multiple) reversal, (B) episodic-like memory with two different time windows, each consisting of an acquisition and retrieval phase, (C) working memory, including two days of clock-wise and two days of counter clock-wise patrolling. The overview is representative for one group, white circles indicating access to water, grey circles indicating blocked access. Each challenge is followed by one day of extinction (not shown) with unrestricted access to liquids. Figure from Gastaldi, Hindermann & Wilke et al. 2025.

**Figure 4:**
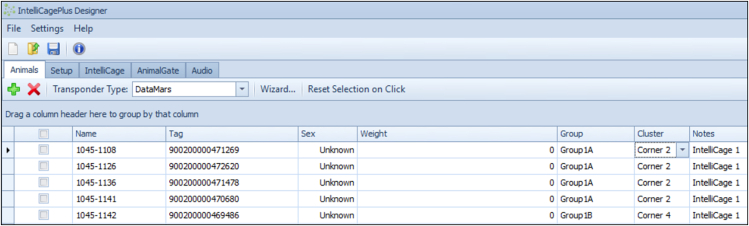
Animal information Animal information have to be entered for each experiment’s designer file under “Animals”. Lab mouse ID (“Name”) and the corresponding transponder ID (“Tag”) are crucial. Sex and weight are optional and not required to run the experiment. Each mouse needs to be assigned one of four groups per IntelliCage (“Group”) and clusters (“Cluster”). The column “Notes” can be used to note the IntelliCage, the mouse was assigned to.

**Figure 5:**
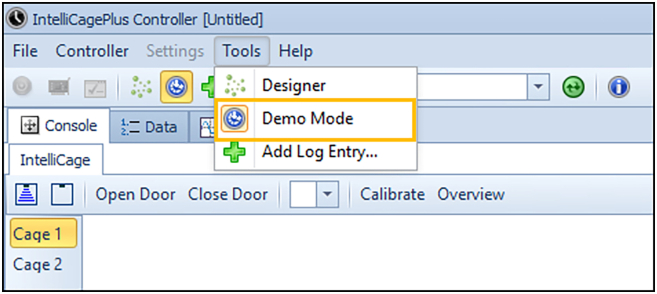
Demo mode to test the IntelliCage system After loading your experimental designer file into the IntelliCagePlus Controller, it is highly recommended to perform the Demo Mode (orange rectangle) for 12–24h prior to your next experiment. This will check all system functions in a random order and generate a final log file, once this mode is stopped.

**Figure 6:**
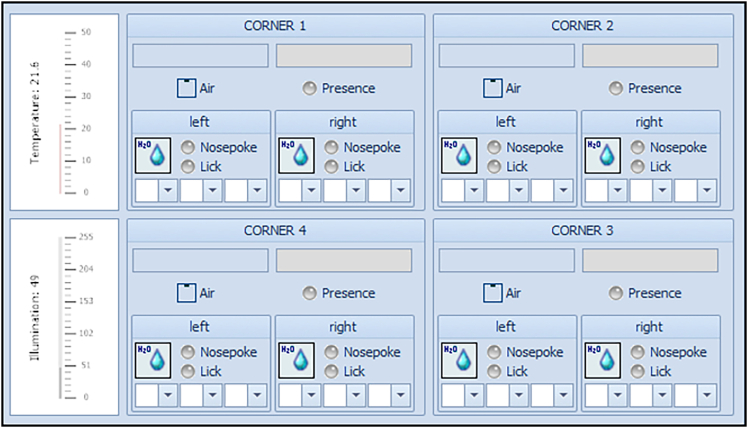
Overview of corner functions in IntelliCagePlus Controller After finishing the Demo Mode, the experimental file can be started for a test to get the overview of corner functions. With the dummy previously added to the animal list, these functions can now be tested for visits (=”Presence”, rectangle will light up green), nosepokes (circle next to “Nosepoke” will light up) and licks (circle next to “Lick” will light up).

3. Preparation of experiment.
  a. Preparation of experimental IntelliCage designer files (for the actual design of the experimental files, please see Gastaldi, Hindermann & Wilke et al. 2025).
    i. Download IntelliR from github (https://github.com/vgastaldi/IntelliR), so you get access to all the required files this protocol is referring to.
    ii. Open the experimental designer file (i.e., “challenge1.experiment”, Figure 3) with IntelliCagePlus Designer.
    iii. Choose “Animals” (Figure 4) and enter the mouse ID’s (“Name”) and respective transponder ID’s (“Tag”).
    iv. Assign a group A-D to each animal (“Group”, e.g., “Group1A”), the number refers to the IntelliCage system connected to the controlling computer.
    v. Assign an operant chamber (=corner, 1–4) to each animal (“Cluster”).
      ***Note:*** Entering sex and body weight is not required to run the experiment. Under “Notes”, you can enter the respective IntelliCage system this animal will be housed in. You can add (green plus) or delete (red cross) lines (Figure 4).
      ***Note:*** In order to enable the system as well as the running designer file to be tested before starting the experiment, it is highly recommended to add one “dummy” per IntelliCage system, which is a not-implanted transponder.
      ***Note:*** Co-housing experimental and control mice together is usually fine and reduces potential bias from micro-environmental variations between cages. However, this strategy may introduce co-learning and care-taking effects, or aggressive behavior against handicapped mice. The choice is best made based on prior knowledge about the mouse strain, disease phenotype, and experimental design.
      **CRITICAL:** Transponder ID’s have to be entered completely and correctly, otherwise the individual animal will neither be tracked nor allowed access to water.
      **CRITICAL:** Whenever possible, corner assignments should be randomized and balanced. Follow ARRIVE guidelines.
    vi. Safe the file and (if applicable) transfer it to the computer controlling the IntelliCage(s).
  b. Preparation of IntelliCage system.
    i. Clean the IntelliCage thoroughly with 70% ethanol and always wear gloves when touching it.
    ii. Before inserting the IntelliCage system into the cage, carefully check all the door mechanisms manually to ensure smooth and quiet movement.
      ***Note:*** In case a door opens and closes loudly or is entirely blocked, it can be disassembled, thoroughly cleaned and re-assembled again.
    iii. Start the IntelliCagePlus Controller software.
    iv. Run “Demo Mode” (Figure 5), the system will now test all functions until you stop this mode.
      ***Note:*** It is highly recommended to test the system for at least 12 h, ideally 24 h.
      **CRITICAL:** Make sure to synchronize the computer time with the time switches controlling the light/dark cycle.
    v. Check the system report after stopping Demo Mode. If there are no error indications, you can continue with the preparation.
    vi. Use IntelliCagePlus Controller to load an experimental file (button beneath “File”, e.g., “challenge2.experiment”, Figure 3).
      ***Note:*** The overview for all four corners and their readouts (Figure 6) should be visualized automatically.
    vii. Start the experimental file by clicking the “Start” button on the far right of the user interface.
    viii. Test the functionality of the operant chambers.
      Insert the dummy transponder together with your index finger into the conditioning corner tube to check if each corner detects a “visit”
      ***Note:*** Visits, rectangle above “Presence” turns green)
      After presence is detected, gently tap both operant walls (=doors) with your index finger.
      ***Note:*** The corner overview at your screen should indicate this by flashing the “Nosepoke” option in the corresponding corner
      Once the door opens, gently tap the bottom tip of the water bottle.
      ***Note:*** Successful “lick” detection should be indicated in the “Lick” option in the corresponding corner on your screen.
      Repeat this for all four corners with both doors and bottles per corner.
      **CRITICAL:** If during this test your presence in the tube is not registered, your hand might be too cold. If the flashing of the “Lick” option does not occur, try again after removing your glove.
    ix. Click the “Stop” button on the far right of the user interface, to end the test session.
  c. Preparation of cage.
    i. Gently lower the IntelliCage into a large cage.
      ***Note:*** Check connections and be careful not to jam any cables
    ii. Add bedding, e.g., 3L wood chip bedding or equivalent.
    iii. Add food and fill up the bottles.
    iv. Add enrichment, i.e., 4× red triangular mouse houses (ACRE011, Tecniplast).
      ***Note:*** There always need to be multiple layers of food pellets, since the mice rely on the lower layer to be pinned down by the other layers. It is necessary to place the houses beneath the feed rack, since it is otherwise too high for the mice to reach. We furthermore recommend to test the water bottles after filling, by gently tapping on the tip.
      ***Note:*** For enrichment, we recommend mouse houses and wood chip bedding rather than paper nesting material. Generally, enrichment that can be dragged into the conditioning corners should be avoided.

## Key resources table

**Table:** 

REAGENT or RESOURCE	SOURCE	IDENTIFIER
**Experimental models: Organisms/strains**		

Wildtype or GMO mice	Institutional or commercial sources. We obtained mice from the Max Planck Institute for Multidisciplinary Sciences	Strain, genotype, sex, and age should be selected based on study design.

**Software and algorithms**		

IntelliR	Gastaldi et al.^1^	https://doi.org/10.5281/zenodo.14912688 and https://github.com/vgastaldi/IntelliR
Designer files challenges	Gastaldi et al.^1^	https://github.com/vgastaldi/IntelliR
R 4.3.0	R Foundation	RRID:SCR_001905
shiny v.1.7.5	Chang et al.^9^	https://shiny.posit.co/; RRID:SCR_001626
ggplot2 3.4.3	Wickham^10^	https://ggplot2.tidyverse.org/; RRID:SCR_014601
car 3.1-2	Fox and Weisberg^11^	https://r-forge.r-project.org/projects/car/; RRID:SCR_022137
effectsize 0.8.6	Ben-Shachar et al.^12^	https://easystats.github.io/effectsize/
rstatix 0.7.2	Kassambara^13^	https://rpkgs.datanovia.com/rstatix/; RRID:SCR_021240
dunn.test 1.35	Dinno^14^	https://CRAN.R-project.org/package=dunn.test
coin 1.4-2	Hothorn et al.^15^	https://coin.r-forge.r-project.org/
multcomp 1.4-25	Hothorn et al.^16^	https://multcomp.r-forge.r-project.org/; RRID:SCR_018255
emmeans v.1.8.8	Lenth^17^	https://rvlenth.github.io/emmeans/; RRID:SCR_018734
FIJI/ImageJ	Schindelin^18^	https://imagej.net/software/fiji/; RRID:SCR_002285

**Other**		

IntelliCage	TSE Systems (transponders can also be purchased from other providers)	RRID:SCR_017404
Mouse houses	Tecniplast	ACRE011 (LWH in mm: 150 × 110 × 77)
RFID Transponders	TSE Systems	PM162-8
Type IV cages	Tecniplast	55 × 38.5 × 20.5 cm
Suture material polypropylene 3-0		

## Step-by-step method details

### Executing and controlling the IntelliCage experiment

**Timing: 15 days**

In this section, longitudinal data for several mouse behaviors will be collected.

**Figure 7:**
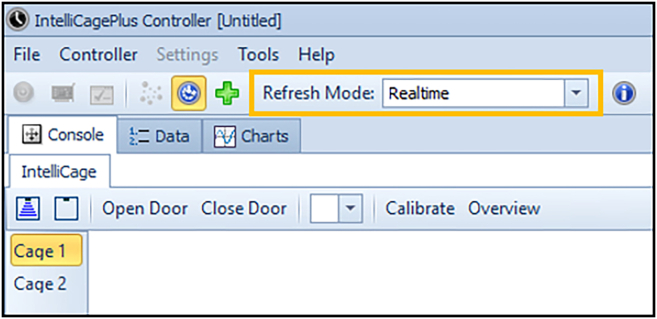
Realtime refresh mode of the IntelliCagePlus controller For the experiments, it is recommended to switch the refresh mode to real-time (orange rectangle), enabling the real-time control of individual liquid consumption.

**Figure 8:**
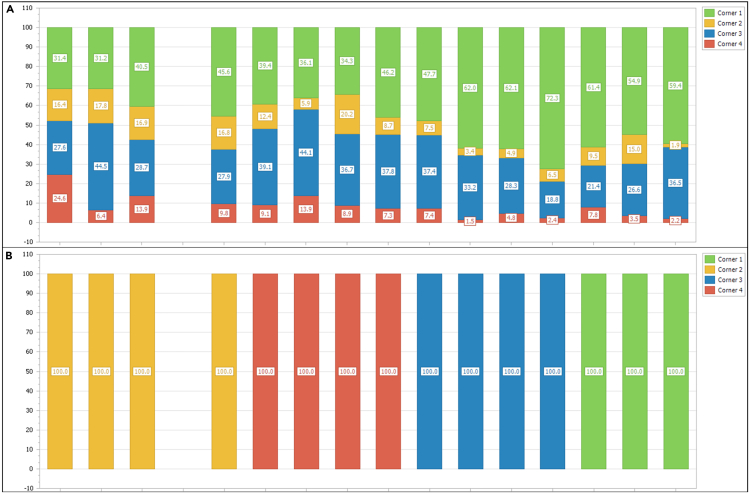
Ratio of licks per corner during habituation and place learning Example data for the comparison of the ratio of individual licks per corner during (A) habituation and (B) place learning, enabling control of correct function of the respective challenge, as well as individual liquid consumption, when absolute values are used. The fourth slot is occupied by the dummy.

**Figure 9:**
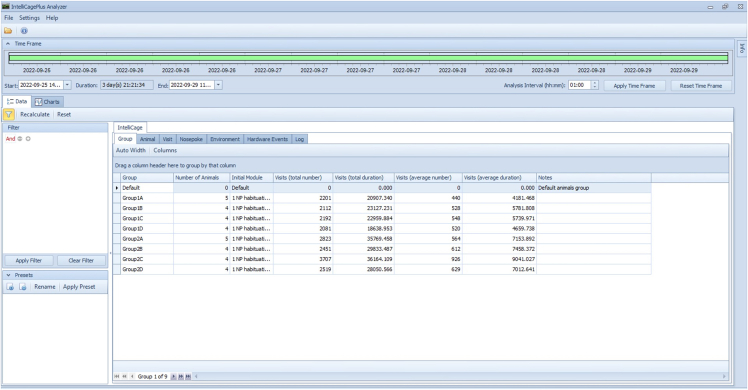
Exporting data via IntelliCageAnalyzer After loading the zip folder with the raw data provided by the IntelliCage system, the time window of interest can be selected by either click and drag the time bar (green) or enter specific date and time in the white boxes below (“Start:”, “End:”). Once selected, click “Apply Time Frame” on the right side below the green bar to apply your chosen time window to all parameters. Select “file” and “Exporting Data…” to choose a folder to export the then created 8.txt files.

**Figure 10:**
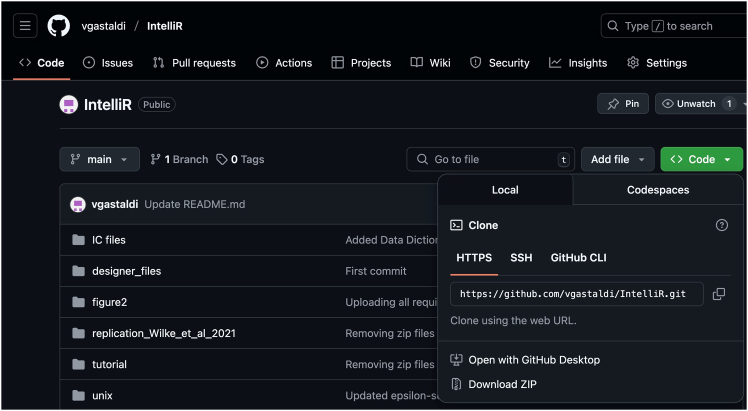
Downloading IntelliR through GitHub The download can be started by clicking on the green button with “< > Code” written on it. A menu will open and you should then click on “Download ZIP”. The downloaded file can be uncompressed and used directly afterwards.

1. Starting the experiment.
  a. Load the experimental designer file (i.e., “challenge3.experiment, Figure 3) into the IntelliCagePlus Controller.
  b. Take the body weight and check transponder of each mouse.
  c. Execute the experimental file and transfer mice into IntelliCage.
2. Running and controlling the experiment.
  a. Use IntelliCagePlus Controller to set Refresh Mode to “Realtime” (Figure 7), which enables you to control the mice’s performance in real time.
  b. Switch to “Charts”, “Preferences” and select “Licks” to see the individual liquid consumption in real time (Figure 8).
    **CRITICAL:** Turn off the screen after using it, if the computer controlling the system is located in the same room as the IntelliCage. Cover all unwanted light sources, such as stand-by indicators, to avoid stress by illumination during the dark phase of your mice.
  c. Use this chart to daily check on the mice’s liquid consumption and corner preferences (Figure 8).
    ***Note:*** Keep interferences to a minimum. Entries to the experimental room should be limited to daily health checks, which should be performed in the inactive phase.
    **CRITICAL:** If a mouse does not consume enough liquid, transfer it back to its original home cage with food and water *ad libitum*. We recommend to not exceed 2 active phases with less than 100 licks. Depending on your experiment, the animal might get re-introduced to the IntelliCage at a later time point.
  d. Remove mice from the IntelliCage and assess their health status and body weight after completion of each challenge.
  e. Clean cages, load the next experimental designer file, and return mice to the IntelliCages.
    ***Note:*** Every provided challenge (Figure 3) starts and ends with a habituation/extinction phase, in which all visited corners give access to liquids without limitation. These phases are used to switch to the next experimental file as well as, if desired, to check the animal’s health status and clean the cage.
3. Ending the experiment.
  a. Use the “Stop” button at the far right of the IntelliCagePlus Controller interface to end the experimental challenge.
  b. Copy the automatically created results folder from the previously determined path to a computer where the data analysis will be performed.
  c. Take the body weight of the mice and transfer them back to their original home cage with food and water *ad libitum.*
  d. Thoroughly clean the IntelliCage.
4. Exporting data.
  a. Open IntelliCageAnalyzer.
  b. Load zip folder (from step 3b) which was automatically created by IntelliCage system (name of the folder: “year-month-day time” when the protocol was stopped).
  c. Select the time window of interest by either click and drag the green time bar or enter specific date and time below (“Start:” & “End:”, Figure 9).
  d. Once time window of interest is selected, click “Apply Time Frame” in order to adjust all parameters accordingly.
  e. Click “file” and “Export Data…” and choose a folder to export the data.
    ***Note:*** IntelliCageAnalyzer will now create 8 .txt files (Animal, Environment, Group, HardwareEvent, Log, Nosepoke, Sessions, Visit), of which the Visit and Nosepoke file are required to successfully execute IntelliR.
  f. Copy the files into folders named Challenge_1, Challenge_2, and Challenge_3, with these three folders placed in another with your project name.
    ***Note:*** Example in Figure 10 named longDTA. This is necessary as IntelliR expects this structure.

### Data analysis via IntelliR

**Timing: 30 min**

After finishing this section, the IntelliCage dataset produced following the previous sections will be fully analyzed, with several spreadsheet files containing the processed data and a folder with figures illustrating the statistical analysis done.

***Note:*** IntelliR is a collection of scripts fully written in the R programming language and does not require any other software apart from R itself and a number of packages that are installed automatically. However, it might be more convenient for some users if they use an Integrated Development Environment (IDE), such as RStudio.

The main download location for IntelliR is through its GitHub page. GitHub is a platform that allows software to be both publicly shared and for users to interact with developers. In addition to being able to reach out to the technical contacts listed below, you can also open issues on GitHub to directly receive support or discuss possible features. All IntelliR scripts have a last modified date on the top of code which can be used to identify your current version and should be provided in case you need support. We are committed to support IntelliR and recommend to always use the latest available version.

5. Installing R.
  a. Download R directly from the Comprehensive R Archive Network: https://cran.r-project.org/mirrors.html.
    ***Note:*** IntelliR was tested using R. 4.3.0 to R 4.4.3, but there is no reason it should not run with more recent versions.
  b. Follow the on-screen instructions to install R in your operating system.
    ***Note:*** We recommend the usage of an IDE such as RStudio. The captured images in this tutorial are from RStudio version 2024.12.1+563 using the “Sky” theme. RStudio can be downloaded from https://posit.co/download/rstudio-desktop/.
6. Downloading IntelliR.
  a. Download IntelliR through its GitHub page: https://github.com/vgastaldi/IntelliR. Figure 10 shows how to download IntelliR through GitHub.

***Note:*** IntelliR was originally deposited in Zenodo under the following DOI: https://doi.org/10.5281/zenodo.14912687. However, since then IntelliR has already been updated and we recommend usage of the latest available version.

**Figure 11:**
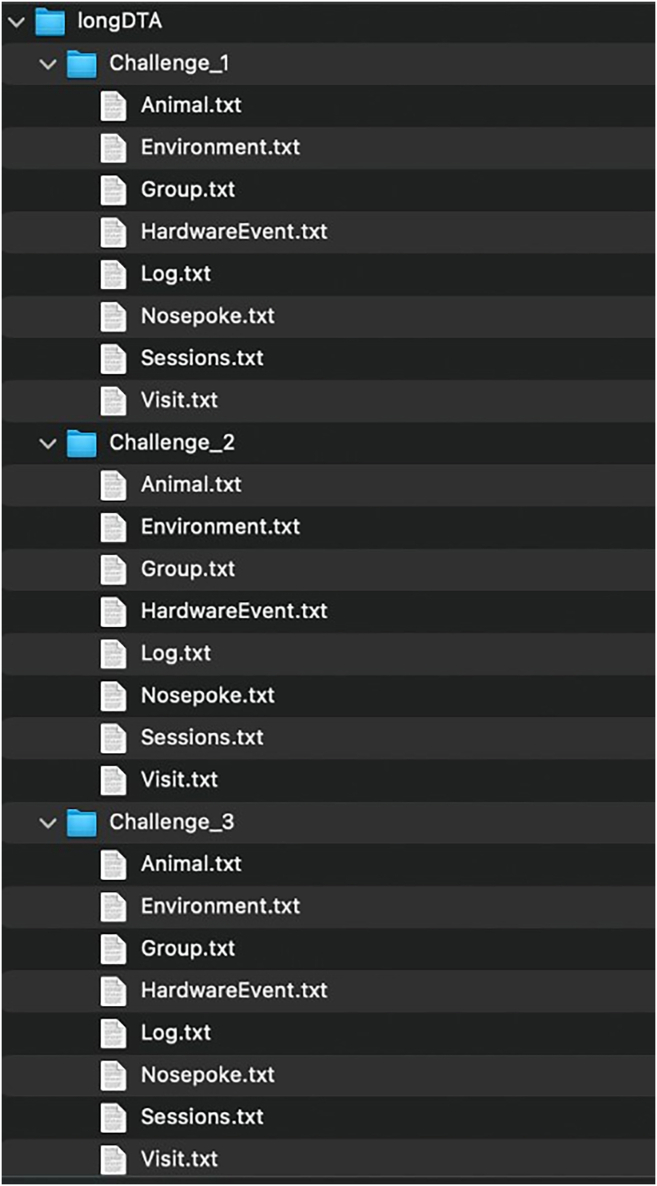
Data structure required by IntelliR The output zip files from step 4f should be placed in folders matching the structure described here. The project folder name should match the name that will be used in the next steps.

**Figure 12:**
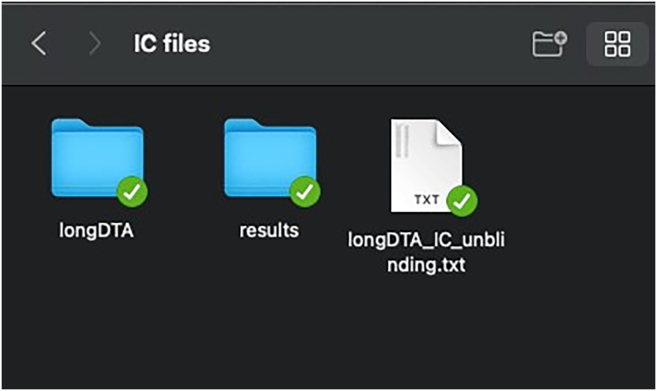
Example folder structure for IntelliR IntelliR works better following the above structure, where a main folder (IC files) contains project folders (here represented by the longDTA folder), a folder for the results produced by IntelliR (here simply named results), and the unblinding files in the root folder (here a single file corresponding to the longDTA project).

**Figure 13:**
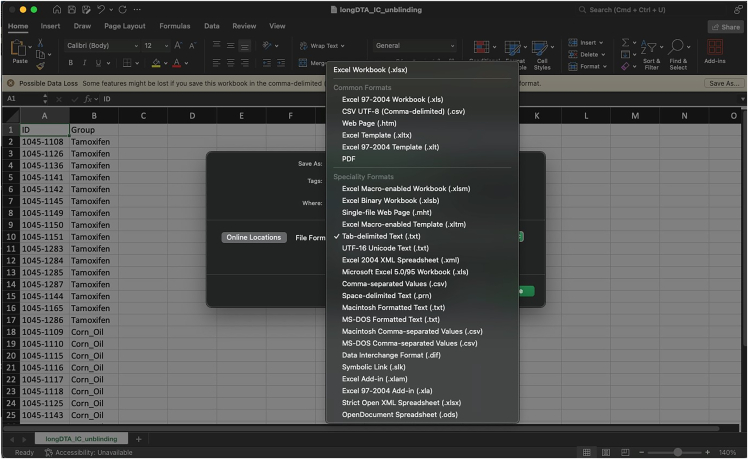
How to save an unblinding file for IntelliR IntelliR requires an unblinding tab-separated .txt file. The file should contain two columns, ID and Group, with the same IDs the animals had in the IntelliCages. This file can be easily made using a spreadsheet software like Excel.

**Figure 14:**
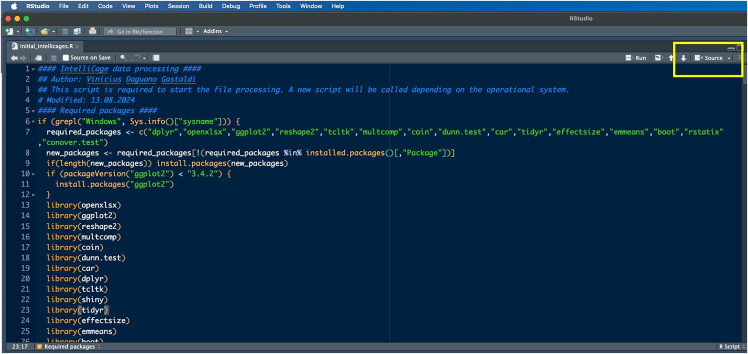
Running IntelliR through RStudio After opening the script with RStudio, click the “Source” button as indicated by the yellow box. Otherwise you can also run the script directly row by row.

**Figure 15:**
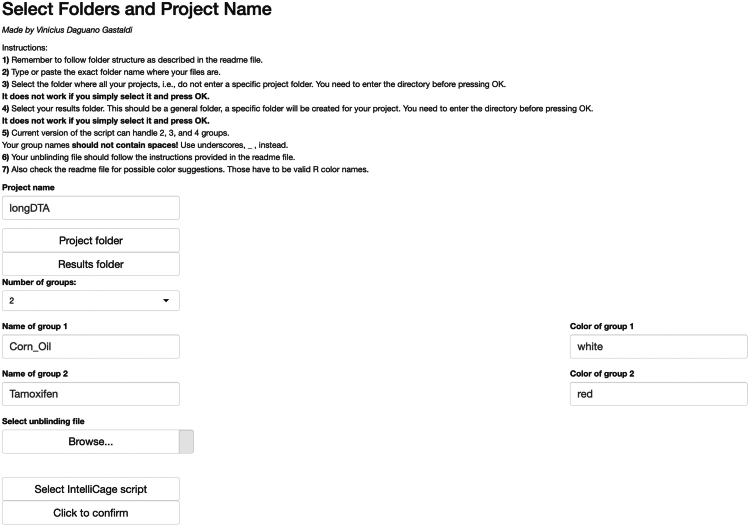
Setting up IntelliR After running IntelliR the shiny webpage above will automatically open. Fill it with your experimental details and select the folders, unblinding file, and processing_intellicages script that matches your operating system. Here we filled it to match our longDTA dataset.

7. Running IntelliR.
  a. Create the specific folder structure required by IntelliR as exemplified in item 4g from the previous section and shown in Figure 11.
    ***Note:*** The file you downloaded from GitHub already contains a complete example structure inside the folder “IC files”. We recommend that you create a specific folder (e.g., IC files) and place all your IntelliCage outputs in project.
  b. Create a folder where the results of IntelliR will be stored as shown in Figure 12.
    ***Note:*** The unblinding file shown in Figure 12 contains the group assignments of the animals that were in the IntelliCages and is needed for you to be able to run IntelliR. The unblinding file has to be a tab separated.txt file with the headers “ID” and “Group”. A simple way to make this file is to use Excel or your preferred spreadsheet software (Figure 13).
  c. Extract the ZIP file from step 2 and you can directly run IntelliR. Both Windows and UNIX based systems can also be directly opened or run the initial_intellicages.R script to start IntelliR.
  d. Open initial_intellicages.R and click on "Source" as shown by the yellow box in Figure 14. A shiny page will open in your default browser.
    ***Note:*** In this tutorial we assume that you are using IntelliR through RStudio, if not, you can simply start it with *Rscript initial_intellicages.R* or *source(“path/to/script/initial_intellicages.R”)* if you have R GUI open.
  e. Fill the newly opened page following the instructions on the top of the page.
    ***Note:*** IntelliR currently supports analysis of two, three, or four groups. In Figure 15 we provide an example on how to fill the page using our “longDTA” dataset. Colors can be chosen either using standard names (as supported by R) or hex codes. When asked to select a IntelliCage script, your selection has to match your operating system (processing_intellicages_windows.R or processing_intellicages_unix.R).
  f. After completely filling the page, click on the button “Click to confirm” and IntelliR will start running (approximatelly 10 minutes).
    **CRITICAL:** We have had reports of issues using the buttons in the interactive page used by IntelliR, mainly from Windows users. Please check Problem 8 in the troubleshooting section.
    ***Note:*** A new folder with your project name will be created in your results folder and the data will be ready for downstream analysis. Additionally, a complete dataset is provided in the “IC files” folder that can be used as an example on how to organize your data and unblinding files.

## Expected outcomes

As described in the last step of the methods section, after around 10 minutes, a new folder for your project will be generated in your results folder. IntelliR processes the IntelliCage data for the challenges described here into 464 variables and includes a complete statistical analysis comparing your groups as described in Gastaldi, Hindermann, Wilke et al. 2025.^1^ A subfolder includes figures for these comparisons for easy visualization. A total of 8 XLSX files are created with their descriptions available in Table 1.

**Table 1:** Description of the output XLSX files created by IntelliR

File name	Description
all_comparisons_set_EM.xlsx	Estimated marginal means (EMMs) values for the learning curve of each one of the challenges
all_comparisons_set.xlsx	Pairwise comparisons for the EMMs described above
corner_preference_ICs_”project”.xlsx	Corner preference for each mouse across all challenges
corner_statistics_”project”.xlsx	Friedman test results for corner preference between groups across all challenges
processedICs_allData_”project”.xlsx	Processed IntelliCage for all individuals across all challenges for a total of 464 variables
processedICs_droppedIndividuals_”project”.xlsx	List of individuals that were removed from the experiment (usually the ones that failed to drink)
processedICs_removedNonDrinkers_”project”.xlsx	Processed IntelliCage for individuals that took part in all challenges for a total of 464 variables
statistics_”project”.xlsx	Statistical analysis for all 464 variables from the processedICs file.

## Quantification and statistical analysis

As described above, IntelliR provides a complete statistical analysis for all calculated variables. A description of the statistical analysis is provided in Gastaldi, Hindermann, Wilke et al. 2025.^1^

## Limitations

This protocol’s limitations can be distinguished between animal-related and technical.

The here presented methodical approach was tested in female mice. As known, male mice, even if grouped since weaning, might develop aggressive behavior towards each other at a certain age and are subsequently required to be separated, which means exclusion from the Intellicage-based experiments. Furthermore, specific mouse models of aging or disease might include reduced motivation and/or capability to access water, hence leading to exclusion from the experiment, too. In general, insufficient water consumption (humane endpoint needs to be determined empirically) may cause individual removal from the experiment.

The current IntelliR version can only automatically process data for the challenges described in this protocol. We have designed a small tutorial with examples on how to repurpose IntelliR for different IntelliCage challenges/protocols which is available in the “tutorial” folder (https://github.com/vgastaldi/IntelliR/tree/main/tutorial). The statistical analysis portion of the pipeline is compatible with any dataset where the first column contains identification values (column name ID), the second column group names (column name Group), and from the third column onwards the variables to be analyzed.

## Troubleshooting

### Problem 1

Infighting of male mice, despite continuous cohousing since weaning (preparation - step 1).

### Potential solution

Utilize adequate handling and housing strategies, i.e., avoid full cage changes and retain scent familiarity, minimize handling stress, provide enrichment (some enrichment like shelters may promote dominance behavior and may need to be tested first), remove the aggressor, reduce group size. If possible, conduct the experiment in young adults. In our experience, 2–6 months old male C57B6 mice can be routinely tested in groups of 16 mice if grouped together upon weaning.

### Problem 2

Transponder cannot be scanned the day after placement or later (preparation - step 2).

### Potential solution

The individual mouse itself or more likely a conspecific within the group caused the loss of the transponder. If it aligns with the experimental schedule, a second intervention for the transponder placement can be done.

### Problem 3

No registered licks for an individual mouse (execution - step 2).

### Potential solution

First, check if there are registered visits for the affected individual. If this is not the case, take the mouse out of the IntelliCage and scan for the transponder. If none can be detected, the animal lost its transponder and needs to be excluded from the experiment. Put it back in the home cage with food and water *ad libitum*. If there is a transponder ID detected, thoroughly compare it with the respective ID entered in the experimental designer file. If the entered ID is incorrect and the currently running protocol is still in the habituation phase, stop the experiment, correct the ID, and start the experiment anew. If the transponder ID can be detected, was correctly entered, and the individual visits are registered, check which group the individual animal was assigned to and subsequently which corners should be free to give access to liquids.

### Problem 4

No licks at one corner despite multiple nosepokes and visits (execution – step 2).

### Potential solution

Check the error log for potential causes. Test the door function with the dummy transponder. Detach, clean, and reattach the door, sometimes bedding can block the opening/closing mechanics. If cleaning includes the use of chemicals (e.g., ethanol), we recommend to also clean the other corners the same way, to avoid place preference/avoidance in your mice.

### Problem 5

Licks at a blocked corner (execution – step 2).

### Potential solution

Check the error log for potential causes. Test the door function with the dummy transponder. Detach, clean, and reattach the door, sometimes bedding can block the opening/closing mechanics. When reattaching the door, heed the position of the magnets.

### Problem 6

Strong corner preference during habituation (execution – step 2).

### Potential solution

Make sure to balance and randomize the corner assignments to reduce bias from initial corner preferences. If possible, avoid assigning this corner as starting corner in tests like place learning. Check the experimental room for potential confounders and remove them, e.g., light sources that stay on during the dark phase, noise, airflow of room ventilation or odors.

### Problem 7

IntelliR fails to open the starting shiny webpage (data analysis – step 3).

### Potential solution

Please check if all packages and dependencies have been installed. R’s console should give you more details if any problems have occurred with them. In the “initial_intellicages.R” script you can also find a list of packages and their tested versions which can help you find if there are any issues.

### Problem 8

IntelliR shiny webpage opens up, but there are errors when selecting files or trying to use the “Confirm” button (data analysis – step 3).

### Potential solution

We tested IntelliR in a few operational system (Windows 10/11, Mac OS 14/15, Ubuntu 22.04/24.04), but issues might occur in different systems/configurations. Two solutions are available.

• New R installations have not presented any issues with the shiny webpage. To avoid interfering with other projects, we recommend the creation of a new conda environment. This can be done through miniconda, which is available here: https://www.anaconda.com/docs/getting-started/miniconda/main. Miniconda is a package manager that can be used to create custom/isolated environments for working R and other tools. After following the installation guide available in their page, you can use IntelliR again after creating a custom environment using: *conda env create -f/path/to/conda_environment/IntelliR_env.yml.*

Activate the environment using conda activate IntelliR_env and proceed with the analysis using Rscript/path/to/your_OS/initial_intellicages.R.

• Another solution is to load the required packages from “initial_intellicages.R” and fill your projects details using “set_up_IC.R”. After filling your details you can directly use “processing_intellicages_WINDOWS/UNIX.R” and IntelliR should run without issues.

### Problem 9

IntelliR starts to run, but there is an issue with the formatting of the IntelliCage files (data analysis – step 3)

### Potential solution

Please check in this protocol our instructions on how to export the IntelliCage files as a few specific columns are required for IntelliR to run properly. You can also check our “longDTA” dataset to ensure your files follow the same format.

## Resource availability

### Lead contact

Further information and requests for resources and reagents should be directed to and will be fulfilled by the lead contact, Hannelore Ehrenreich (hannelore.ehrenreich@zi-mannheim.de).

### Technical contact

Technical questions on executing this protocol should be directed to and will be answered by the technical contacts, Martin Hindermann (hindermann@mpinat.mpg.de) for questions regarding mouse experiments and Vinicius Daguano Gastaldi (gastaldi@mpinat.mpg.de or through GitHub) for questions regarding IntelliR.

### Materials availability

This study did not generate new unique reagents.

### Data and code availability

The original code and data have been deposited to Zenodo (https://doi.org/10.5281/zenodo.14912688) and GitHub (https://github.com/vgastaldi/IntelliR). See also Gastaldi et al.^1^

## Acknowledgments

This work was supported by the European Research Council (ERC) Advanced Grant to H.E. under the European Union’s Horizon Europe research and innovation program (acronym BREPOCI; grant agreement no. 101054369). Furthermore, the study has been fostered by the Max Planck Society and the Max Planck Förderstiftung. The R logo in the graphical abstract (R logo copyright 2016 The R Foundation; https://www.r-project.org/logo/) is used under CC-BY-SA 4.0 (https://creativecommons.org/licenses/by-sa/4.0/). Changes were made to the original logo. Furthermore, the authors thank TSE Systems, Berlin, for their support.

## Author contributions

The protocol was conceptualized, designed, and originally published by V.D.G., M.H., J.W., A.R., S.A., S.K., and H.E. The manuscript was written by M.H., V.D.G., and J.W. and edited by all authors. Representative data were generated by M.H., V.D.G., J.W., A.R., S.A., and S.K.

## Declaration of interests

The authors declare no competing interests.
